# Application of Computational Systems Biology to Explore Environmental Toxicity Hazards

**DOI:** 10.1289/ehp.1103533

**Published:** 2011-08-17

**Authors:** Karine Audouze, Philippe Grandjean

**Affiliations:** 1 Center for Biological Sequence Analysis, Department of Systems Biology, Technical University of Denmark, Lyngby, Denmark; 2 Institute of Public Health, University of Southern Denmark, Odense, Denmark; 3 Department of Environmental Health, Harvard School of Public Health, Boston, Massachusetts, USA

**Keywords:** computational biology, DDT, genomics, proteomics, systems biology

## Abstract

Background: Computer-based modeling is part of a new approach to predictive toxicology.

Objectives: We investigated the usefulness of an integrated computational systems biology approach in a case study involving the isomers and metabolites of the pesticide dichlorodiphenyltrichloroethane (DDT) to ascertain their possible links to relevant adverse effects.

Methods: We extracted chemical–protein association networks for each DDT isomer and its metabolites using ChemProt, a disease chemical biology database that includes both binding and gene expression data, and we explored protein–protein interactions using a human interactome network. To identify associated dysfunctions and diseases, we integrated protein–disease annotations into the protein complexes using the Online Mendelian Inheritance in Man database and the Comparative Toxicogenomics Database.

Results: We found 175 human proteins linked to *p,p*´-DDT, and 187 to *o,p*´-DDT.Dichlorodiphenyldichloroethylene (*p,p*´-DDE) was the metabolite with the highest number of links, with 52. We grouped proteins for each compound based on their disease annotations. Although the two data sources differed in linkage to diseases, integrated results predicted that most diseases were linked to the two DDT isomers. Asthma was uniquely linked with *p,p*´*-*DDT, and autism with *o,p*´*-*DDT. Several reproductive and neurobehavioral outcomes and cancer types were linked to all three compounds.

Conclusions: Computer-based modeling relies on available information. Although differences in linkages to proteins may be due to incomplete data, our results appear meaningful and suggest that the parent DDT compounds may be responsible for more disease connections than the metabolites. The findings illustrate the potential use of computational approaches to toxicology.

In its report, *Toxicity Testing in the 21st Century*, the National Research Council called for the development of new approaches to human health risk assessment that would rely, in part, on computer-based models rather than animal testing and epidemiology (National Research Council 2007). Although these recommendations were timely and visionary, progress has been slow, possibly because of the need for elaborate validation of models before the adoption of a new approach to predictive toxicology. However, toxicological databases and computational methods have developed further and now seem to be even better suited for applications in environmental health research. Through international efforts, publicly available databases have been combined and fine-tuned to provide linked information regarding chemical names, synonyms, chemical structures, hazards, chemical exposures, and potential risks to human health within several categories: acute, developmental toxicity, reproductive toxicity, and cancer (Judson et al. 2008). As a complement to these databases, ChemProt, a new disease chemical biology database (Center for Biological Sequence Analysis 2011), provides information on chemical links to proteins along with chemical names, chemical structures, and diseases (Taboureau et al. 2011). These data resources can be used to develop computational models for predicting toxicological end points or possible biological mechanisms.

Various models already have been developed, most of them structure-based. Chemicals are grouped according to their similar structural features or fragments that can be related to a particular toxicity end point, for example, ToxMatch (Pavan and Worth 2008) and Derek Nexus (Lhasa Limited 2011), or by using quantitative structure–activity relationships approaches, as in Computer Assisted Evaluation of Industrial Chemical Substances According to Regulations (CAESAR) (Cassano et al. 2010). In parallel, systems chemical biology has emerged as a field that integrates chemical information with biological databases (Oprea et al. 2007). It has therefore become possible to generate a generic computational systems biology model that aims at revealing the underlying molecular mechanisms of xenobiotics and the biological pathways they may disrupt (Audouze et al. 2010). In this approach, toxicogenomics data are combined with systems biology information to provide a high-confidence human protein–protein association network. We previously used this integrative systems biology method to decipher unexpected relationships between di(2-ethylhexyl)phthalate and gamma-aminobutyric acid receptors (Audouze et al. 2010). Similarly, Gohlke et al. (2009) developed a network of complex diseases to integrate molecular pathways associated with both genetic and environmental factors. However, these computational predictions and others like them must be interpreted in light of the caveat that the databases used contain available information only and that metabolism and other factors may affect the chemical–protein interactions. Given the new developments and our promising initial experience, we have attempted to illustrate the current potential of advanced computational systems biology to assess the potential hazards associated with a group of environmental chemicals with substantial, although incomplete, toxicological and epidemiological information.

The pesticide dichlorodiphenyltrichloroethane (DDT) was marketed as an ideal insecticide with long-term protection; however, the environmental persistence of DDT and its metabolite dichlorodiphenyldichloroethylene (DDE) resulted in bioaccumulation and delayed or latent adverse effects. Experimental studies have explored mainly the effects of DDT dosages, whereas epidemiology research primarily has used the serum concentration of the DDE metabolite as an exposure biomarker. The DDE concentration may reflect, to some degree, past exposures to the parent DDT compound that had been metabolized later, but it may also originate directly from DDE residues in food. Thus, concentrations present at the time of blood sampling may represent neither the correct chemical species nor the active dose present at the time when a possible adverse effect was initiated. This conundrum is difficult to resolve from epidemiology data alone, and the experimental data do not cover all the potential outcomes for the relevant isomers and metabolites [Agency for Toxic Substances and Disease Registry (ATSDR) 2002]. We therefore chose to carry out computational chemistry analyses of the DDT compounds from a human systems biology perspective. Our dual purpose was to obtain new information that might link relevant outcomes to specific DDT compounds and to ascertain to what degree the currently available data sources would allow such analyses using a systems chemical biology approach. Although this study was not intended as a formal validation study, we consider a family of compounds for which extensive although somewhat equivocal epidemiological and experimental evidence exists, with the result that our analysis includes suspected causal agents and anticipated negative controls.

## Methods

**Figure 1:**
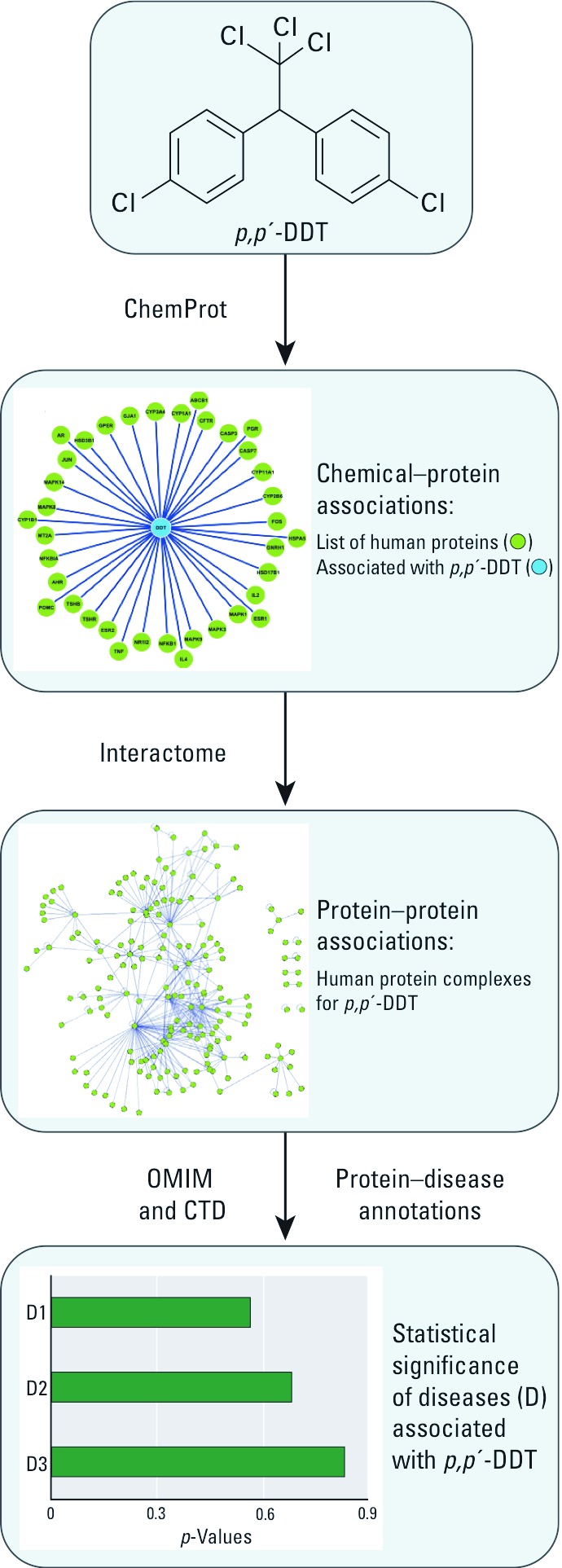
Overview of the systems chemical biology three-step approach. (1) Extraction of existing knowledge using a disease chemical biology database (ChemProt) to generate chemical–protein networks for *p,p*´-DDT. (2) Creation of protein complexes by protein enrichment using a high-confidence set of experimental protein–protein interactions. (3) Statistical ranking of diseases (D) known or predicted to be linked to *p,p*´-DDT after integration of protein–disease annotations to protein complexes based on information in the Online Mendelian Inheritance in Man (OMIM) database and the Comparative Toxicogenomics Database (CTD).

Using data from the ToxProfile of the ATSDR (2002) with the 2008 appendix and literature searches of PubMed, we chose to concentrate on inflammatory, reproductive and endocrine, neurobehavioral, and malignant diseases. DDT isomers and metabolites were examined by multi-step data integration in the systems chemical biology approach (Figure 1). In short, in the first step, we extracted existing knowledge from a disease chemical biology database to generate compound-specific human protein networks. In the second step, protein enrichment, we used a high-confidence set of experimental human protein–protein interactions to identify protein complexes. In the final step, we ranked the diseases predicted to be linked to the DDT compounds using an integration of protein–disease annotations in the protein complexes.

More specifically, first we extracted the human protein–chemical associations from the newly established disease chemical biology database, ChemProt (Taboureau et al. 2011), a compilation of multiple chemical–protein annotation resources that contains 700,000 unique chemicals connected with 30,578 proteins. This database assembles chemical–protein connections from multiple sources, such as ChEMBL (de Matos et al. 2010), BindingDB (Liu et al. 2007), PDSP Ki database (Roth et al. 2004), and PubChem bioassays (Wheeler et al. 2007). The integrated associations comprise both binding and separate gene expression data, as the deregulation of a gene by a chemical may not be due necessarily to a physical interaction between the compound and the protein, e.g., in the form of binding, but could entail a response at a cellular level. ChemProt can predict uncharacterized chemical–protein interactions based on the similar structure of compound pairs. In the present study, only chemical–protein relationships with experimental support were kept for the analysis, while otherwise unsupported, predicted connections were disregarded.

Second, we explored protein–protein interactions through an in-house human interactome network based on experimental data from humans and 21 model organisms (Lage et al. 2007, 2008). Using a probabilistic confidence scoring scheme, all interactions in the human interactome have been validated against a gold standard (Rual et al. 2005). We used an updated version of the protein–protein interaction network consisting of refined experimental proteomics data (Lage et al. 2010). The current interactome contains 507,142 unique protein–protein interactions (PPIs). These data are derived from sources such as the Biomolecular Interaction Network Database (BIND; Bader et al. 2003), Biological General Repository for Interaction Datasets (BioGRID; Stark et al. 2006), Molecular INTeraction (MINT) database (Zanzoni et al. 2002), DIP_FULL dataset (Salwinski et al. 2004), Human Protein Reference Database (HPRD; Mishra et al. 2006), IntAct (an open source molecular interaction database) (Hermjakob et al. 2004), Mammalian Protein–Protein Interaction (MMPI) database (Pagel et al. 2005), MPact [the Munich Information Center for Protein Sequences (MIPS) protein interaction resource on yeast] (Guldener et al. 2006), Reactome (Joshi-Tope et al. 2005), and Kyoto Encyclopedia of Genes and Genomes (KEGG) databases (Kanehisa et al. 2006). Data are transferred between organisms by using the InParanoid Eukaryotic Ortholog Groups’ database (InParanoid 2009; O’Brien et al. 2005). Among the PPIs included, 414,543 are listed at least once as interactions between two human proteins. The human interactome includes 22,997 genes. Among the gene products, 14,441 human proteins have known interactions with other proteins; the others are singletons. In addition to direct interactions between known proteins, we also included indirect interactions by considering proteins involved in common pathways. This step used a statistical procedure shown to provide optimal network significance (Lage et al. 2010). As a result, the list of relevant proteins associated with each compound is extended by inclusion of their known first-order protein–protein interaction partners and other proteins participating in the same pathways.

Third, to identify diseases or dysfunctions associated with the specific chemicals examined (i.e., *p,p*´*-*DDT, *o,p*´*-*DDT, and *p,p*´*-*DDE), we integrated protein-specific information related to their involvement in disease pathogenesis with the proteins identified. The Online Mendelian Inheritance in Man (OMIM) database [Hamosh et al. 2005; National Center for Biotechnology Information (NCBI) 2011b] and the Comparative Toxicogenomics Database (CTD 2011; Davis et al. 2011) were selected as the most useful sources of protein–disease information. The OMIM database is a highly reliable compilation of genetic variants from medical and genetics publications. The CTD database contains both direct (manually curated) and inferred gene–disease relationships and may therefore provide less certain associations. Whereas direct associations derive from experimental models or epidemiological studies, inferred relationships are established through indirect evidence. Thus, if gene A is associated with disease B, and gene A has a curated interaction with chemical C, then chemical C has a curated relationship with disease B (Davis et al. 2011). These inferred relationships are more explorative and allow hypothesis generation using protein–disease connections in the chemical space.

The different strengths of the two databases, OMIM and CTD, are illustrated by comparison of the data sets. The OMIM (version 2009) contains 3,748 connections involving 2,728 unique diseases, while the CTD (version 2009) contains 252,056 (hypothetical) protein–disease associations involving 2,580 unique diseases. We translated diseases having a Medical Subject Heading identification (MeSH ID) number in CTD into OMIM ID numbers to the extent possible, given that a single MeSH ID number may correspond to several OMIM ID numbers. Based on OMIM ID numbers, 1,934 diseases are shared between the two databases, 2,042 proteins are common to both, and they correspond to 2,528 shared protein–disease associations of 255,804 interactions listed. Because of the limited overlap, we used both resources to explore the predictions in terms of protein–disease relationships and biological confidence with regard to grouping proteins within a binary metric distance (Audouze et al. 2010).

Using R software (R Development Core Team 2010), *p*-values were calculated for each group of proteins, assuming a hypergeometric distribution. To take into account the large numbers of potential protein–disease connections in the CTD, we performed Bonferroni adjustments of all *p*-values for protein clusters based on the CTD. For this source of information, we chose a classical family-wise error rate of 0.05 as cutoff to adjust *p*-values. We mapped all human proteins identified to EntrezGene (NCBI 2011a) identifiers using the Clone/GeneID converter (Alibes et al. 2007).

## Results

Using the ChemProt database, we extracted 38 relevant human proteins for *p,p*´*-*DDT, 83 for *o,p*´*-*DDT, and 18 for *p,p*´*-*DDE [for details, see Supplemental Material, Table 1 (http://dx.doi.org/10.1289/ehp.1103533)]. We used the resulting three lists of proteins independently to create three human protein networks. For *o,p*´*-*DDE, only 11 proteins were identified, and all had already been identified earlier as targets for *p,p*´-DDE. Likewise, *p,p*´*-*DDD was connected with only 10 proteins, and *o,p*´*-*DDD with only three [progesterone receptor, androgen receptor, and estrogen receptor 1, the latter two overlapping with *p,p*´*-*DDD]. No information was obtained on the methylsulfonyl metabolites. Our data analysis therefore concentrated on the three substances first mentioned.

**Table 1:** Number of proteins associated with *p,p*´*-*DDT, *o,p*´*-*DDT, and *p,p´-*DDE within the different steps of the systems biology procedure.

Number of proteins				
Chemical name	ChemProt	Interactome (PPIs)	Mapped to EntrezGene
*p,p*´*-*DDT		38		182 (381)		175
*o,p*´*-*DDT		83		189 (235)		187
*p,p*´*-*DDE		18		56 (92)		52

**Figure 2:**
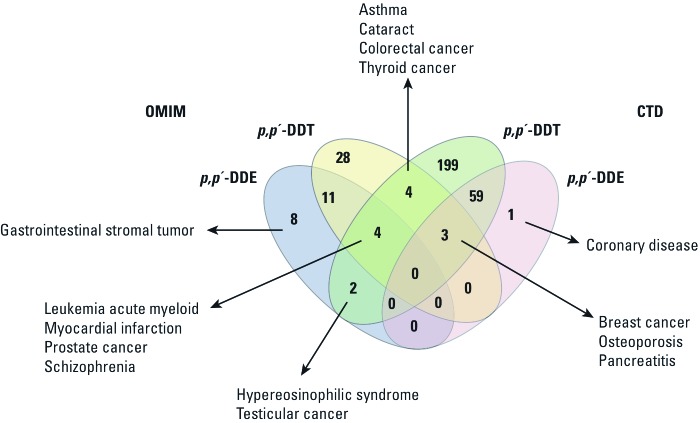
Venn diagram showing the number of diseases overlapping between *p,p*´*-*DDE and *p,p*´*-*DDT, using disease annotations extracted from the OMIM and the CTD databases. Data on *o,p*´*-*DDT are not shown, as most of its disease links overlapped with *p,p*´*-*DDT.

We generated a human protein network for each chemical by determining protein–protein interaction partners associated with each protein network. Proteins with a GeneEntrez ID were retained. We identified 175 proteins for the *p,p*´*-*DDT analysis, 187 proteins for *o,p*´*-*DDT, and 52 proteins for *p,p*´*-*DDE (Table 1). Disease enrichment based on OMIM and CTD protein–disease annotations led to identification of diseases associated with each chemical (Table 2). Some diseases were not significantly associated with a chemical (e.g., when linked only via one protein). As anticipated, the CTD provided many more potential disease associations than the OMIM. This difference between the OMIM- and CTD-based predictions likely reflects the extent of uncertain and incomplete evidence within the two data sources. The specific and overlapping disease annotations for *p,p*´-DDT and *p,p*´*-*DDE within disease clusters for the two different data sources are shown in Figure 2. Most of the diseases predicted for *o,p*´*-*DDT overlapped with predictions for *p,p*´*-*DDT. Overall, 35 diseases (based on OMIM data) and 210 diseases (based on CTD data) appeared to be unique for *p,p*´*-*DDT. All OMIM-based predictions had higher *p*-values than CTD-based predictions, but these differences should be interpreted in light of the fewer known proteins in the OMIM database associated with the diseases predicted. For example, asthma was connected with *p,p*´-DDT via a single protein, tumor necrosis factor, in the OMIM database (*p* = 0.147), whereas CTD information predicted asthma via connections through 48 proteins (*p* = 0.002) [for details, see Supplemental Material, Table 2 (http://dx.doi.org/10.1289/ehp.1103533)]. In contrast, only a few diseases appeared to be unique for *p,p*´*-*DDE: 1 based on CTD data (i.e., coronary heart disease), and 8 based on OMIM data.

**Table 2:** Number of diseases associated with *p,p*´*-*DDT, *o,p*´*-*DDT, and *p,p*´-DDE using the OMIM and the CTD databases.

Chemical name	OMIM	CTD
*p,p*´*-*DDT		50		271
*o,p*´*-*DDT		45		77
*p,p*´*-*DDE		25		62

**Figure 3:**
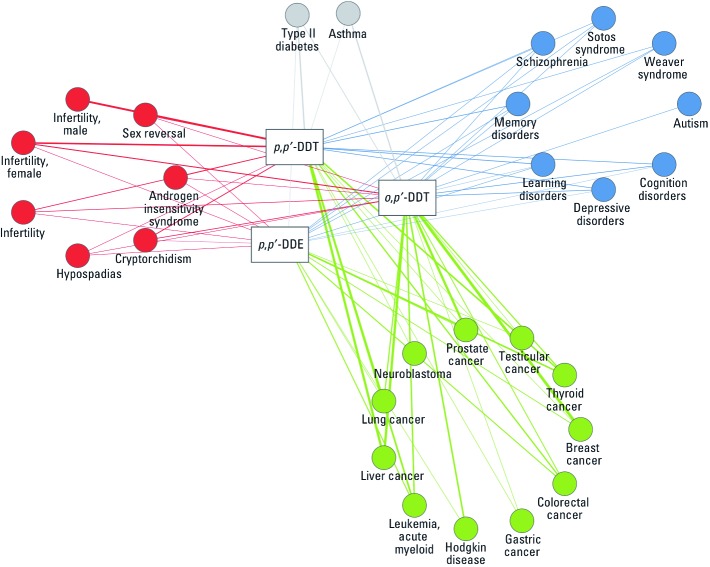
Disease–chemical associations network. The circles represent diseases, with colors representing phenotype categories: red, reproductive disorders; blue, neurodevelopmental-related diseases; green, cancers; gray, other diseases. Rectangles represent the three chemicals studied. The heavier the weight of the connecting lines, the greater the number of proteins linking a chemical to a disease (determined using the OMIM and CTD databases and ChemProt as resources).

To synthesize this information, we focused on the four major categories of disease phenotypes previously linked to DDT exposures: inflammatory, reproductive and endocrine, neurobehavioral, and carcinogenic (Figure 3). [See Supplemental Material, Table 2 (http://dx.doi.org/10.1289/ehp.1103533) for details on the *p*-values (adjusted when based on CTD data), sources of protein–disease information (CTD or OMIM), and specific genes linked to the proteins.] Depending on the source of protein–disease annotations, the phenotypes may be more or less specific, but all annotations identified in the databases were kept to avoid any subjective judgment in data extraction. As expected, the predictions varied somewhat between the two databases, OMIM and CTD.

Overall, of 175 proteins linked to *p,p*´-DDT after exploration of the human interactome in the CTD, only 29 were retrieved using the OMIM database. Among those 29 proteins, one was associated with asthma among the total of 13 connected with this disease in the OMIM database (which contains a total of 2,387 proteins in the current version). Of the 131 proteins associated with *p,p*´-DDT in the CTD, 48 had associations with asthma (of a total of 1,977 proteins potentially linked to asthma among the 10,509 proteins included in the CTD). The asthma linkage was found only with regard to *p,p*´*-*DDT; no relationships were found for *p,p*´*-*DDE, and the single link to *o,p*´*-*DDT had a nonsignificant *p*-value.

Several links to reproductive phenotypes were retrieved. Female infertility disorders representing diminished ability or inability of a woman to achieve conception, although not further specified in the resources used, were connected with the three chemicals. In parallel, male reproductive disorders, including hypospadias and cryptorchidism, were predicted for *p,p*´*-*DDT, *o,p*´*-*DDT, and *p,p*´-DDE. Genes linked to these male phenotypes included the androgen receptor listed for hypospadias. Although more genes were identified from the CTD, the statistical significance decreased. For cryptorchidism, both the androgen receptor and gonadotropin-releasing hormone 1 were predicted. The identities of some of the genes [for details, see Supplemental Material, Table 2 (http://dx.doi.org/10.1289/ehp.1103533)] suggested that the DDTs may also have other endocrine-disrupting effects in humans. Type 2 diabetes mellitus appeared to be connected with all three substances via 9 proteins for *p,p*´*-*DDE (nonsignificant *p*), 40 proteins for *p,p*´*-*DDT (*p* < 0.0001), and 25 proteins for *o,p*´*-*DDT (nonsignificant *p*).

For neurobehavioral diseases, Soto and Weaver syndromes were predicted with low *p*-values via the nuclear receptor binding SET domain protein 1 based on OMIM data for all three substances. When using the CTD, additional disorders were identified, including learning disorders (*p* = 0.01 for 7 proteins for *p,p*´*-*DDE; *p* = 0.013 for 12 proteins for *o,p*´*-*DDT; and *p* < 0.0001 for 25 proteins for *p,p*´*-*DDT). Similar results were obtained for memory disorders (*p* = 0.05 for 6 proteins for *p,p*´*-*DDE; and *p* < 0.0001 for 23 proteins for *p,p*´*-*DDT). These results again reflect the diversity of information present in the two data sources used. Interestingly, *o,p*´*-*DDT was the only substance studied that predicted a linkage with autism, via the hepatocyte growth factor receptor.

Various cancers appeared to be connected with all three substances. Breast cancer was predicted for all three DDTs using CTD data and for both parent DDTs using OMIM data (where the link for *p,p*´*-*DDT is via a single protein, the hyaluronan-mediated motility receptor, *p* = 0.208). Using the CTD, the link for *p,p*´*-*DDT involved 22 proteins (*p* < 0.001), and for *p,p*´*-*DDE, 24 proteins (*p* = 0.021), again reflecting the larger amount of information on these chemicals in the CTD. For information on other cancers, see Supplemental Material, Table 2 (http://dx.doi.org/10.1289/ehp.1103533).

## Discussion

The discipline of systems chemical biology combines experimental findings with computational models with the aim of understanding the effect of xenobiotics on a biological system. This field of research now allows integration of disparate information sources such as high confidence protein–protein association data, protein–disease annotations, “omics” information, and other biological data from databases to explore hidden and unknown connections. Recent advances in toxicogenomics also contribute information to the impact of small molecules on genes and proteins. Because of these developments, advanced computational systems chemical biology models have been developed to decipher the association between environmental chemicals and diseases (Audouze et al. 2010). Accordingly, the combined sources of information can now be applied in computational models to predict associations between chemical exposures and human health effects. Although such *in silico* prediction of course cannot be considered a proof of causal links, it nonetheless provides justification for hypothesis generation and contributes to interpreting toxicology information from other sources.

The integration of chemical biology and systems biology in a systems chemical biology approach is unsupervised and is based entirely on known chemical and biological information about the behavior of xenobiotics, their interactions with specific proteins, and the consequences in regard to protein–protein interactions and possible disease pathogenesis in humans. The statistical procedure helps in ranking the interactions, but it may not reflect dose–effect relationships. Thus, the disease annotations identified by this *in silico* approach represent hypothetical causal links that need to be explored and verified in a biological setting, whether *in vitro* or *in vivo*, with the aim of deciphering potential toxicity and modes of action of the chemicals. In addition, the links identified are based on current information, which is incomplete, and the absence of a link therefore does not necessarily mean lack of plausibility regarding a particular adverse effect. This problem is clearly illustrated by comparing the predictions generated by using the two databases, with the greater number of predicted associations found using the CTD, which includes less-certain, indirect evidence. Although the smaller number of associations suggested by the OMIM database may be better documented, this likely represents an underestimation because of incomplete information. Although substantial overlap was detected, neither of these databases can therefore be considered definitive, and they may not become so in the foreseeable future. Thus, all predictions depend on current knowledge, much of which is yet uncertain. And again, dose–response relationships cannot be inferred from the predictions. Although these limitations do not invalidate the results, the caveats are important when interpreting the findings. In particular, linkage to asthma and autism, for example, should not be interpreted as indicating that DDT may be a specific cause of these diseases, which are likely multicausal.

In this study, we chose to use the computational model for a major pesticide to assess the usefulness of the computational approach as a tool to bridge gaps in our understanding of environmentally related disease processes by identifying potential mechanistic links. The main results are in close accordance with toxicology findings and results from prospective epidemiological studies that relied on serum concentrations at relevant time windows.

In regard to asthma, the two data sources agree to a substantial extent that the disease may be uniquely associated with the parent DDT compounds. Only nonallergic disease in adult farmers has been linked to DDT usage (Hoppin et al. 2009). In children at 6 years of age, the presence of asthma—independent of atopy—was linked to increased concentrations of *p,p*´*-*DDE found in umbilical cord serum (Sunyer et al. 2006). Our findings suggest that the epidemiological linkage to *p,p*´*-*DDE may be indirect and that future studies should also assess exposures to the parent compound.

Antiandrogenic effects of *p,p*´*-*DDT and *p,p*´*-*DDE have been demonstrated experimentally (Gray et al. 2001). One epidemiological study showed no association between the anogenital distance in boys and *p,p*´*-*DDT and *p,p*´*-*DDE concentrations in maternal serum (Longnecker et al. 2007), whereas another study found a significant association for the latter (Torres-Sanchez et al. 2008). It is not clear from this evidence whether *o,p*´*-*DDT plays any role in this respect. Most studies on cryptorchidism and hypospadias had limited statistical power or focused only on *p,p*´*-*DDE (Brucker-Davis et al. 2008). In regard to semen quality, a cross-sectional study of pesticide sprayers currently using DDT showed inverse associations with the current serum concentration, especially for *p,p*´*-*DDT (Aneck-Hahn et al. 2007), whereas another study involving infertile men showed that the sum of all *p,p*´ isomers was negatively associated with sperm concentration (Messaros et al. 2009). As for antiandrogenic effects studies, much additional information is available on *p,p*´*-*DDE. Likewise, in regard to type 2 diabetes, epidemiological studies have generally focused on *p,p*´*-*DDE (ATSDR 2002), but our data suggest that *p,p*´-DDT may be a more likely etiologic agent.

Experimental animal studies document that *p,p*´-DDT is a neurotoxicant, but evidence on other DDT isomers and metabolites is less extensive (ATSDR 2002). A prospective human study in California suggested that the maternal serum concentration of *p,p*´-DDT during pregnancy was a stronger predictor of the neurodevelopment of the child up to 12 months of age than were the *p,p*´-DDE and *o,p*´-DDT concentrations (Eskenazi et al. 2006). Similarly, a study in Spain showed that neuropsychological performance at 4 years of age decreased in children with higher *p,p*´-DDT concentrations in umbilical cord serum (Ribas-Fito et al. 2006). Other studies relied solely on *p,p*´-DDE concentrations (Darvill et al. 2000; Gladen and Rogan 1991; Rogan and Gladen 1991; Sagiv et al. 2008) and may therefore have missed effects associated with the parent compound.

There is sufficient evidence for carcinogenicity of DDT in animals (ATSDR 2002). The main cancer form studied in regard to human DDT exposure is breast cancer. Perhaps the strongest evidence comes from the prospective follow-up of women who provided a blood sample in connection with the Child Health and Development Studies in California in 1959–1967, where 129 women subsequently developed breast cancer before 50 years of age. The odds ratios showed a significant association with *p,p*´*-*DDT, but not with *o,p*´*-*DDT or *p,p*´*-*DDE (Cohn et al. 2007). Support for this notion comes from a Danish study that relied on a 17-year follow-up from 1976: An increased odds ratio for breast cancer was found among women with the highest quartile of serum *p,p*´-DDT concentrations, whereas this tendency was not seen for *p,p*´-DDE (Hoyer et al. 1998). Unfortunately, the majority of studies in this field have relied on DDE measurements, often in cross-sectional designs. Evidence for liver cancer (McGlynn et al. 2006) and testicular cancer (Cohn et al. 2010; Purdue et al. 2009) also supports the notion that *p,p*´*-*DDT may be the major carcinogen, as associations with *p,p*´*-*DDE could be due to breakdown of the parent compound. For example, in a case–control study of non-Hodgkin lymphoma using concurrent serum samples, a significant association was seen with *p,p*´-DDE, but the high frequency of nondetectable *p,p*´-DDT concentrations did not allow a proper comparison (Spinelli et al. 2007).

In light of the toxicological and epidemiological evidence on adverse health effects of DDT compounds, this study shows that the *in silico* approach is highly relevant and meaningful. That said, a major problem in the epidemiological literature is that it mainly links serum concentrations of *p,p*´*-*DDE to suspected adverse effects. As some of these effects may be due rather to *p,p*´*-*DDT, the studies therefore rely on a proxy variable for past DDT exposure. However, one cannot assume that all *p,p*´*-*DDE originates from the subject’s own breakdown of the parent compound, and the serum-DDE concentration is therefore imprecise and may be biased. The degree of imprecision will likely vary with the age and time of exposure. Such exposure misclassification generally leads to an underestimation of the true effect of the substances studied. If the effects are ascribed to the unmeasured parent compound, toxicokinetic calculations may perhaps be applied to generate a more appropriate exposure measure that reflects the exposure to the active substance. The opposite error may play a role in toxicological studies, where effects have been attributed to the parent compound, although potentially mediated through a metabolite. However, our results suggest that this possibility is of little significance in regard to DDT.

Overall, our findings demonstrate that the systems chemical biology approach is feasible and may have a pivotal role in considering potential causal associations derived from toxicology and epidemiology studies. Although our approach is based on the current knowledge base and may therefore have overlooked some linkages, the results show that the DDT compounds examined, while chemically related, have tertiary structures, gene expression profiling, and binding properties that deviate sufficiently from one another to predict outcomes that differ substantially. The differences in predicted outcomes are not likely to be due to differences in the amount of information available. Thus, the major parent compound, *p,p*´*-*DDT, would seem to be much more potent in regard to adverse effects than its isomers and metabolites. In addition, we have identified several new potential target diseases not hitherto examined as relevant outcomes [see Supplemental Material, Table 2 (http://dx.doi.org/10.1289/ehp.1103533)]. These potential targets deserve attention in future experimental and epidemiological studies to provide a more complete basis for risk assessment.

The usefulness and validity of the computational approach is likely to improve as more information becomes available, including more chemical–protein data as well as data from “omics”and gene–environment interaction studies. Furthermore, the results of the disease–chemical association analysis will improve in the future as newer, more complete, and curated data become available to expand and fine-tune our understanding of protein–disease associations. In addition, studies like this one will contribute to the necessary validation of *in silico* approaches and findings, and cumulated experience will help in interpreting such analyses in light of possible unknown interactions and absent dose–effect relationships. Thus, the results obtained with the DDT compounds serve as an illustration of the potential use of computational predictions in toxicology, epidemiology, and environmental health research. The visions expressed by the National Research Council committee on transforming toxicology therefore seem reasonable and realistic.

## Supplemental Material

(229 KB) PDF
