# Supplementary material for: Application of Computational Systems Biology to Explore Environmental Toxicity Hazards
Source: Environ Health Perspect. 2011 Aug 17;119(12):1754–9. doi: 10.1289/ehp.1103533 (PMC3261980; doi:10.1289/ehp.1103533)
Supplement: (229 KB) PDF [file ehp.1103533.s001.pdf]

SUPPLEMENTARY MATERIAL

Application of Computational Systems Biology to Explore Environmental Toxicity Hazards

By Karine Audouze[1] and Philippe Grandjean[2,3]

[1]Center for Biological Sequence Analysis, Department of Systems Biology, Technical University of Denmark, Lyngby, Denmark

[2]Institute of Public Health, University of Southern Denmark, Odense, Denmark; and

[3]Department of Environmental Health, Harvard School of Public Health, Boston, MA, USA

Corresponding author:

Karine Audouze, PhD, Center for Biological Sequence Analysis, Department of Systems Biology, Technical University of Denmark, DK-2800 Lyngby, Denmark.

Phone: +45-45252472. Fax: +45-45931585. E-mail: karine@cbs.dtu.dk

Contents

Supplementary Material, Table 1. List of human chemical-protein associations extracted from ChemProt. (pp. 2-5)

Supplementary Material, Table 2. List of inflammatory, reproductive, neurodevelopmental and cancer diseases predicted to be linked to chemicals using CTD (*p*-value adjusted for multiple comparisons) and OMIM (unadjusted *p*-value). All associations with *p*-values superior to 0.5 are show with the number of gene/protein., where the source corresponds to the database used for the prediction: min ids are OMIM ids and mesh ids are from CTD. (pp. 6-12)

Supplementary Material, Table 1: List of human chemical-protein associations extracted from ChemProt.

**Chemical name**	**Gene name**
DDE	*ABCB1*
DDE	*ACVR1C*
DDE	*ACVRL1*
DDE	*AHR*
DDE	*ALK*
DDE	*AR*
DDE	*CYP19A1*
DDE	*CYP1B1*
DDE	*ERBB3*
DDE	*ESR1*
DDE	*ESR2*
DDE	*FSHB*
DDE	*IL4*
DDE	*KIT*
DDE	*PDGFRA*
DDE	*PDGFRB*
DDE	*PGR*
DDE	*SRD5A2*
DDT	*ABCB1*
DDT	*AHR*
DDT	*AR*
DDT	*CASP3*
DDT	*CASP7*
DDT	*CFTR*
DDT	*CYP11A1*
DDT	*CYP1A1*
DDT	*CYP1B1*
DDT	*CYP2B6*
DDT	*CYP3A4*
DDT	*ESR1*
DDT	*ESR2*
DDT	*FOS*
DDT	*GJA1*
DDT	*GNRH1*
DDT	*GPER*
DDT	*HSD17B1*
DDT	*HSD3B1*
DDT	*HSPA5*
DDT	*IL2*

Supplementary Material, Table 1 (continued)

**Chemical name**	***Gene name***
DDT	*IL4*
DDT	*JUN*
DDT	*MAPK1*
DDT	*MAPK14*
DDT	*MAPK3*
DDT	*MAPK8*
DDT	*MAPK9*
DDT	*MT2A*
DDT	*NFKB1*
DDT	*NR1I2*
DDT	*PGR*
DDT	*POMC*
DDT	*RXRB*
DDT	*TNF*
DDT	*TSHR*
o,p′-DDT	*ADAMTS9*
o,p′-DDT	*ANAPC1*
o,p′-DDT	*AR*
o,p′-DDT	*ASRGL1*
o,p′-DDT	*BCL2*
o,p′-DDT	*BNIP1*
o,p′-DDT	*C14ORF138*
o,p′-DDT	*C6ORF48*
o,p′-DDT	*CABLES1*
o,p′-DDT	*CAPN9*
o,p′-DDT	*CARS*
o,p′-DDT	*CHAC2*
o,p′-DDT	*CIRBP*
o,p′-DDT	*CYP2B6*
o,p′-DDT	*CYP3A11*
o,p′-DDT	*CYP3A4*
o,p′-DDT	*DHRS3*
o,p′-DDT	*EDG3*
o,p′-DDT	*EIF2B4*
o,p′-DDT	*ERBB2*
o,p′-DDT	*ERCC3*
o,p′-DDT	*ESR1*
o,p′-DDT	*ESR2*
o,p′-DDT	*ETNK2*
o,p′-DDT	*FAM107A*
o,p′-DDT	*FOLR1*

Supplementary Material, Table 1 (continued)

**Chemical name**	***Gene name***
o,p´-DDT	*FOXD1*
o,p´-DDT	*G0S2*
o,p´-DDT	*GALNT4*
o,p´-DDT	*GALR2*
o,p´-DDT	*GDPD5*
o,p´-DDT	*GPR142*
o,p´-DDT	*HAPLN3*
o,p´-DDT	*HES2*
o,p´-DDT	*HIST1H2BD*
o,p´-DDT	*HIVEP1*
o,p´-DDT	*HMGN2*
o,p´-DDT	*IMPDH2*
o,p´-DDT	*KCTD20*
o,p´-DDT	*KRT17*
o,p´-DDT	*LPPR2*
o,p´-DDT	*MAGEA3*
o,p´-DDT	*MANSC1*
o,p´-DDT	*MAPK1*
o,p´-DDT	*MAPK13*
o,p´-DDT	*MAPK3*
o,p´-DDT	*MEOX1*
o,p´-DDT	*MET*
o,p´-DDT	*MFGE8*
o,p´-DDT	*MFN2*
o,p´-DDT	*MLPH*
o,p´-DDT	*MRPS7*
o,p´-DDT	*NEIL2*
o,p´-DDT	*NR1I2*
o,p´-DDT	*OLFM1*
o,p´-DDT	*OLFML3*
o,p´-DDT	*PDCD7*
o,p´-DDT	*PELI1*
o,p´-DDT	*PGR*
o,p´-DDT	*PI4KA*
o,p´-DDT	*PIM1*
o,p´-DDT	*PKM2*
o,p´-DDT	*PLA2G3*
o,p´-DDT	*PRKAR2A*
o,p´-DDT	*RBM13*
o,p´-DDT	*SCAND2*

Supplementary Material, Table 1 (continued)

**Chemical name**	***Gene name***
o,p´-DDT	*SF3B5*
o,p´-DDT	*SHBG*
o,p´-DDT	*SIAH2*
o,p´-DDT	*SLC1A5*
o,p´-DDT	*SLC31A1*
o,p´-DDT	*SLCO2A1*
o,p´-DDT	*SQLE*
o,p´-DDT	*TGFA*
o,p´-DDT	*TGFB2*
o,p´-DDT	*THBS1*
o,p´-DDT	*TMED4*
o,p´-DDT	*TMEM16A*
o,p´-DDT	*TSC22D1*
o,p´-DDT	*TSPAN12*
o,p´-DDT	*TXNIP*
o,p´-DDT	*ZKSCAN2*
o,p´-DDT	*ZMYM3*

Supplementary Material, Table 2. List of inflammatory, reproductive, neurodevelopmental and cancer diseases predicted to be linked to chemicals using CTD (*p*-value adjusted for multiple comparisons) and OMIM (unadjusted *p*-value). All associations with *p*-values superior to 0.5 are shown with the number of gene/protein., where the source corresponds to the database used for the prediction: min ids are OMIM ids and mesh ids are from CTD.

DISEASE	CHEMICAL	NUMBER OF GENES	SOURCE	P-VAL	LIST OF GENES
ANDROGEN INSENSITIVITY SYNDROME	DDE	1	mim:300068	0.00628	*AR*
ANDROGEN INSENSITIVITY SYNDROME	opDDT	1	mim:300068	0.0109	*AR*
ANDROGEN INSENSITIVITY SYNDROME	DDT	1	mim:312300	0.0121	*AR*
ASTHMA	DDT	48	mesh :d001249	0.0028122	*CFTR;PNRC1;DUSP10;MAPK14;CYP3A4;CYP11A1;TSC22D3;DUSP1; DUSP5;DUSP6;NR0B1;AHR;ERF;ESR1;ETV5;FOS;FOSL2;GJA1;GNRHR; HAL;HSD17B1;IL1B;IL2;AR;JUN;MT2A;NFATC4;NFKB1;NRD1;NTRK3; ABCB1;PLA2G2A;POMC;POU4F1;POU4F2;MAPK1;MAPK3;MAPK8; MAPK9;RAGE;RXRB;TNF;TSHB;CASP3;PLA2G10;CASP7;NR0B2;NR1I2*
ASTHMA	DDT	1	mim:600807	0.147	*TNF*
AUTISM	opDDT	1	mim:611015	0.0109	*MET*
BREAST CANCER	DDT	22	mesh :d001932	0.00095202	*MAPK14;CYP3A4;CYP11A1;FOS;GNRH1;GNRHR;HSPA5;AR;JUN; MT2A;NFKB1;NUCB2;ABCB1;MAPK1;MAPK3;MAPK8;MAPK9;RXRB; TNF;CASP3;CASP7;NR1I2*
BREAST CANCER	DDE	24	mesh :d001943	0.0214	*PNRC1;CYP27A1;NR0B1;AHR;SPRED2;ERBB3;ESR1;ESR2;ETV5;FGF12; FSHB;AR;KIT;PDGFRA;PDGFRB;PGR;ABCB1;POU4F1;POU4F2;WIPI1; RNF4;PRDM2;NCOA4;NRG2*
BREAST CANCER	opDDT	1	mim:114480	0.189	*HMMR*
BREAST CANCER	DDT	1	mim:114480	0.208	*HMMR*
COGNITION DISORDERS	DDT	22	mesh :d003072	0.00160218	*MAPK14;CYP3A4;DUSP2;DUSP6;ESR1;ESR2;GJA1;GNRH1;HAL;IL1B; AR;PGR;ABCB1;MAPK1;MAPK3;PTPRR;RXRB;TNF;NCOA4;CASP7; LMNB2;TNFSF11*

Supplementary Material, Table 2 (continued)

**DISEASE**	**CHEMICAL**	**NUMBER OF GENES**	**SOURCE**	**P-VAL**	***LIST OF GENES***
COGNITION DISORDERS	opDDT	22	mesh :d003072	0.00160218	*CYP3A4;DUSP2;DUSP6;ERBB2;ESR1;ESR2;HAL;AR;GAL;PGR;PIM1; MAPK1;MAPK3;PTPRR;BCL2;SHBG;MARCKSL1;TGFB2;THBS1;NCOA4; FYTTD1;LMNB2*
COGNITION DISORDERS	DDE	11	mesh :d003072	0.0034056	*CYP27A1;ERBB3;ESR1;ESR2;AR;PDGFRA;PDGFRB;PGR;ABCB1; NCOA4;NRG2*
COLORECTAL CANCER	DDT	40	mesh :d015179	2.14E-07	*DUSP10;CASP12;MAPK14;CYP3A4;CYP11A1;COMMD6;DUSP2;DUSP3; AHR;ESR1;ESR2;FOS;GJA1;GNRHR;HMMR;HSPA5;IL1B;IL2;AR;JUN; JUNB;NFKB1;NUCB2;PGR;ABCB1;PLA2G2A;MAPK1;MAPK3;MAPK8; MAPK9;RNF4;RXRB;TNF;UGT2B7;PRDM2;CASP3;CASP7;BARX2; TNFSF11;NR1I2*
COLORECTAL CANCER	opDDT	1	mim:114500	0.189	*PLA2G2A*
COLORECTAL CANCER	DDT	1	mim:114500	0.208	*PLA2G2A*
COLORECTAL CANCER	opDDT	28	mesh :d015179	0.27864	*TXNIP;AKAP10;CLDN4;CYP3A4;DUSP2;ERBB2;ESR1;ESR2;HMMR; AR;KRT17;BFAR;PGR;PLA2G2A;MAPK1;MAPK3;MAPK13;BCL2; RNF4;SQLE;TGFA;TGFB2;TNFAIP6;UGT2B7;PRDM2;BCL2L14;NR1I2; MFN2*
CRYPTORCHIDISM	DDT	28	mesh :d003456	4.31E-05	*CFTR;CMTM3;CYP11A1;DUSP1;DUSP6;NR0B1;AHR;ESR1;ESR2;FOS; GJA1;HSD17B1;AR;MT2A;NFKB1;PGR;ABCB1;WIPI1;AVPR2;MAPK1; MAPK3;MAPK8;RNF4;NFKBIZ;TNF;TSHR;CASP3;NR1I2*
CRYPTORCHIDISM	DDE	12	mesh :d003456	0.0056244	*NR0B1;AHR;SPRED2;ERBB3;ESR1;ESR2;FSHB;AR;PGR;ABCB1;WIPI1; RNF4*
CRYPTORCHIDISM	opDDT	24	mesh :d003456	0.0086172	*CMTM3;DUSP6;NR0B1;ERBB2;ESR1;ESR2;FMOD;AR;MUC4;GAL;PGR; WIPI1;AVPR2;MAPK1;MAPK3;BCL2;RNF4;SHBG;TGFA;GALR2;TSC22D1; NR1I2;CABLES1;SCARB2*
DEPRESSIVE DISORDERS	DDT	22	mesh :d003865	0.00187566	*MAPK14;CYP3A4;DUSP2;DUSP6;ESR1;ESR2;GJA1;GNRH1;HAL;IL1B; AR;PGR;ABCB1;MAPK1;MAPK3;PTPRR;RXRB;TNF;NCOA4;CASP7; LMNB2;TNFSF11*
DEPRESSIVE DISORDERS	opDDT	22	mesh :d003865	0.00187566	*CYP3A4;DUSP2;DUSP6;ERBB2;ESR1;ESR2;HAL;AR;GAL;PGR;PIM1; MAPK1;MAPK3;PTPRR;BCL2;SHBG;MARCKSL1;TGFB2;THBS1; NCOA4;FYTTD1;LMNB2*

Supplementary Material, Table 2 (continued)

**DISEASE**	**CHEMICAL**	**NUMBER OF GENES**	**SOURCE**	**P-VAL**	***LIST OF GENES***
DEPRESSIVE DISORDERS	DDE	11	mesh :d003865	0.003741	*CYP27A1;ERBB3;ESR1;ESR2;AR;PDGFRA;PDGFRB;PGR;ABCB1; NCOA4;NRG2*
GASTRIC CANCER	opDDT	1	mim:137215	0.123	*ERBB2*
GASTRIC CANCER	DDT	1	mim:137215	0.137	*IL1B*
HODGKIN DISEASE	opDDT	26	mesh :d006689	0.0106812	*CLDN4;CYP3A4;DUSP6;ERBB2;ESR1;ESR2;HNF4G;MET;MT1G;PGR; PIM1;MAPK1;MAPK3;MAPK13;BCL2;NSD1;BLVRA;ANAPC1;TGFB2; THBS1;TNFAIP6;DAP3;LMNB2;NR1I2;EIF2B4;TP53I3*
HODGKIN DISEASE	DDE	11	mesh :d006689	0.150156	*AHR;ERBB3;ESR1;ESR2;FGF21;KIT;PDGFRB;PGR;ABCB1;NSD1;DAP3*
HYPOSPADIAS	DDE	1	mim:300633	0.0125	*AR*
HYPOSPADIAS	opDDT	1	mim:300633	0.0217	*AR*
HYPOSPADIAS	DDT	1	mim:300633	0.0242	*AR*
INFERTILITY	DDT	25	mesh :d007246	0.00036636	*CFTR;CMTM3;CYP11A1;DUSP1;DUSP6;AHR;ESR1;ESR2;FOS;GJA1; HSD17B1;AR;MT2A;NFKB1;PGR;ABCB1;WIPI1;MAPK1;MAPK3; MAPK8;RNF4;NFKBIZ;TNF;CASP3;NR1I2*
INFERTILITY	DDE	11	mesh :d007246	0.0146286	*AHR;SPRED2;ERBB3;ESR1;ESR2;FSHB;AR;PGR;ABCB1;WIPI1;RNF4*
INFERTILITY	opDDT	21	mesh :d007246	0.062694	*CMTM3;DUSP6;ERBB2;ESR1;ESR2;FMOD;AR;MUC4;GAL;PGR; WIPI1;MAPK1;MAPK3;BCL2;RNF4;SHBG;TGFA;TSC22D1;NR1I2; CABLES1;SCARB2*
INFERTILITY, FEMALE	DDT	14	mesh :d007247	1.94E-10	*CYP3A4;ESR1;ESR2;GJA1;IL1B;AR;JUN;NFKB1;PGR;MAPK1;MAPK3; TNF;CASP3;NR1I2*
INFERTILITY, FEMALE	opDDT	10	mesh :d007247	2.61E-05	*CYP3A4;ESR1;ESR2;AR;PGR;MAPK1;MAPK3;BCL2;SHBG;NR1I2*
INFERTILITY, FEMALE	DDE	4	mesh :d007247	0.232716	*ESR1;ESR2;AR;PGR*

Supplementary Material, Table 2 (continued)

**DISEASE**	**CHEMICAL**	**NUMBER OF GENES**	**SOURCE**	**P-VAL**	***LIST OF GENES***
INFERTILITY, MALE	DDT	58	mesh :d007248	0.0097782	*CFTR;MAPK14;CYP3A4;CYP11A1;DUSP1;DUSP6;AHR;ELK3;ESR1; ESR2;NSUN6;FOS;FOSL2;PLA2G2D;GJA1;GNRH1;COMMD5;HAL; HSD17B1;HSPA5;IL1B;IL2;AR;JUN;JUNB;MT2A;NEO1;NFKB1;NTRK3; FKBP7;PGR;ABCB1;PLA2G1B;PLA2G2A;PLA2G5;POMC;POU4F1;MAPK1; MAPK3;MAPK8;MAPK9;STARD7;PTPRR;NFKBIZ;BLVRA;ZFP36L1;TNF; TSHB;TSHR;UGT2B7;CUEDC2;NCOA4;CASP3;PLA2G10;CASP7;NR0B2; CTSF;NR1I2*
LEARNING DISORDERS	DDT	25	mesh :d007859	2.16E-15	*CYP3A4;CYP11A1;ESR1;ESR2;FOS;GJA1;GNRH1;HSPA5;IL1B;AR;JUN; MT2A;NFKB1;NTRK3;PGR;ABCB1;POU4F1;MAPK1;MAPK3;MAPK8; RXRB;TNF;CASP3;CASP7;NR1I2*
LEARNING DISORDERS	DDE	7	mesh :d007859	0.0108876	*ESR1;ESR2;AR;PDGFRA;PGR;ABCB1;POU4F1*
LEARNING DISORDERS	opDDT	12	mesh :d007859	0.0133902	*CYP3A4;ESR1;ESR2;AR;MET;PGR;POU4F1;MAPK1;MAPK3;BCL2;SHBG; NR1I2*
LEUKEMIA, ACUTE MYELOID	DDT	52	mesh :d015470	4.02E-05	*CFTR;PNRC1;DUSP10;MAPK14;CYP3A4;CYP11A1;TSC22D3;DUSP1;DUSP6; NR0B1;AHR;PAOX;ERF;ESR1;ESR2;ETV5;FOS;GJA1;HAL;HSD17B1;HSPA5; IL1B;IL2;AR;JUN;MT2A;NFKB1;NRD1;NTRK3;NUCB2;ABCB1;PLA2G2A; POU4F1;POU4F2;MAPK1;MAPK3;MAPK8;MAPK9;RAGE;RXRB;NSD1; GRAMD3;ZFP36L1;TNF;TSHB;CASP3;PLA2G10;CASP7;NR0B2;KLF11; TNFSF11;NR1I2*
LEUKEMIA, ACUTE MYELOID	DDE	2	mim:601626	0.00593	*KIT;NSD1*
LEUKEMIA, ACUTE MYELOID	opDDT	44	mesh :d015470	0.086172	*TXNIP;PNRC1;CLDN4;CPT1A;CYP3A4;DUSP6;NR0B1;PAOX;ERBB2;ERF; ESR1;ESR2;ETV5;AKR1B1;HAL;IMPDH2;AR;KRT17;MEOX1;MET;MT1G; MUC4;PAX1;PIM1;PKM2;PLA2G2A;POU4F1;POU4F2;MAPK1;MAPK3; RAB27B;BCL2;NSD1;GRAMD3;BTC;TGFA;TGFB2;THBS1;PLA2G10;KLF11; SCEL;NR1I2; TP53I3;MFN2*
LEUKEMIA, ACUTE MYELOID	opDDT	1	mim:601626	0.189	*NSD1*
LEUKEMIA, ACUTE MYELOID	DDT	1	mim:601626	0.208	*NSD1*

Supplementary Material, Table 2 (continued)

DISEASE	CHEMICAL	NUMBER OF GENES	SOURCE	P-VAL	*LIST OF GENES*
LIVER CANCER	opDDT	71	mesh :d008113	0.046698	*APBB3;CORO2B;TXNIP;MAGED2;CMTM3;CLDN4;CPT1A;CYP3A4; DUSP6;PAOX;KRTCAP2;ERBB2;ERCC3;ESR1;ESR2;NSUN6;AKR1B1; FMOD;PLA2G2D;HAL;HNF4G;HPD;AR;KRT17;MET;ASAH1;MT1G; MUC4;GAL;PCTK2;PGR;PIM1;PLA2G2A;WIPI1;ANO1;MAPK1;MAPK3; MAPK13;STARD7;OLFML3;RAB27B;BCL2;RNF4;NSD1;BLVRA;SHBG; ANAPC1;MARCKSL1;GRAMD3;SQLE;TGFA;TGFB2;THBS1;TNFAIP6; UGT2B7;UCK2;VRK2;DAP3;NCOA4;SH3BGRL3;PLA2G10;KLF11;SPSB2; LMNB2;TSC22D1;NR1I2;EIF2B4;CABLES1;SCARB2;TP53I3;MFN2*
LIVER CANCER	DDT	69	mesh :d008113	0.204336	*CFTR;CASP12;CMTM3;MAPK14;PDIK1L;CYP3A4;CYP11A1;TSC22D3; DUSP1;DUSP6;AHR;PAOX;ELK3;ELK4;ESR1;ESR2;NSUN6;FOS;FOSL2; PLA2G2D;GJA1;GNRH1;HAL;HNF4G;HPD;HSD17B1;HSPA5;HTR3A;IL1B; IL2;AR;JUN;JUNB;MT2A;NFATC4;NFKB1;NUCB2;PGR;ABCB1;PLA2G2A; POMC;WIPI1;MAPK1;MAPK3;MAPK8;MAPK9;STARD7;RNF4;NSD1; NFKBIZ;BLVRA;GRAMD3;ZFP36L1;TNF;TSHB;TSHR;UGT2B7;DAP3; NCOA4;CASP3;PLA2G10;CASP7;NR0B2;KLF11;LMNB2;PARG;TNFSF11; CTSF;NR1I2*
LUNG CANCER	DDT	49	mesh :d008175	3.10E-06	*CFTR;DUSP10;MAPK14;CYP3A4;CYP11A1;TSC22D3;DUSP1;DUSP6; AHR;PAOX;ELK3;ELK4;ESR1;ESR2;FOS;FOSL2;GJA1;HNF4G;HSPA5; HTR3A;IL1B;IL2;JUN;JUNB;MT2A;NFATC4;NFKB1;NUCB2;PGR;ABCB1; MAPK1;MAPK3;MAPK8;MAPK9;RXRB;NSD1;BLVRA;GRAMD3;ZFP36L1; TNF;TSHB;DAP3;BIRC7;CASP3;CASP7;KLF11;LMNB2;TNFSF11;NR1I2*
MEMORY DISORDERS	DDT	23	mesh :d008569	1.17E-14	*CFTR;DUSP10;MAPK14;CYP3A4;DUSP1;AHR;ESR1;FOS;IL1B;JUN;NFKB1; NTRK3;ABCB1;POU4F1;MAPK1;MAPK3;MAPK8;ZFP36L1;TNF;BIRC7; CASP3;CASP7;NR1I2*
MEMORY DISORDERS	DDE	6	mesh :d008569	0.05676	*AHR;ERBB3;ESR1;PDGFRA;ABCB1;POU4F1*
NEUROBLASTOMA	DDE	1	mim:256700	0.0249	*ALK*
PROSTATE CANCER	DDT	55	mesh :d010190	0.000106554	*CFTR;PNRC1;DUSP10;MAPK14;CYP3A4;CYP11A1;CYP11B2;TSC22D3; DUSP1;DUSP6;NR0B1;AHR;PAOX;ERF;ESR1;ESR2;ETV5;FOS;FOSL2; GJA1;GNRHR;HAL;HSD17B1;HSPA5;IL1B;IL2;AR;JUN;JUNB;MT2A;NFKB1; NRD1;NTRK3;NUCB2;ABCB1;PLA2G2A;POU4F1;POU4F2;MAPK1;MAPK3; MAPK8;MAPK9;RAGE;RNF4;RXRB;GRAMD3;ZFP36L1;TNF;TSHB;CASP3; PLA2G10;CASP7;NR0B2;KLF11;NR1I2*

Supplementary Material, Table 2 (continued)

DISEASE	CHEMICAL	NUMBER OF GENES	SOURCE	P-VAL	*LIST OF GENES*
PROSTATE CANCER	opDDT	70	mesh :d011471	0.034314	*TXNIP;PNRC1;CMTM3;CLDN4;CPT1A;CYP3A4;DUSP2;DUSP5;DUSP6; DUSP9;ERBB2;ERCC3;ERF;ESR1;ESR2;NSUN6;AKR1B1;FMOD;HAL; HNF4G;HPD;AR;KRT17;MET;MT1F;MT1G;MUC4;GAL;PGR;PIM1;PLA2G1B; WIPI1;NUDT11;MAPK1;MAPK3;MAPK13;OLFML3;PTPRR;BCL2;RNF4; NSD1;BLVRA;SHBG;ANAPC1;SLC1A5;MARCKSL1;BNIP1;SQLE;TGFA; TGFB2;THBS1;TNFAIP6;UGT2B7;PRDM2;DAP3;MLPH;BCL2L14;NCOA4; PLA2G10;FYTTD1;KLF11;LMNB2;SCEL;TSC22D1;NR1I2;EIF2B4;CABLES1; SCARB2;TP53I3;MFN2*
PROSTATE CANCER	DDE	1	mim:176807	0.124	*AR*
PROSTATE CANCER	opDDT	1	mim:176807	0.206	*AR*
PROSTATE CANCER	DDT	1	mim:176807	0.227	*AR*
SCHIZOPHRENIA	DDT	16	mesh :d012559	0.0034056	*CYP11A1;FOS;APOL2;GJA1;GNRH1;IL2;AR;JUN;MT2A;NFKB1;MAPK1; MAPK3;MAPK8;TNF;CASP3;CASP7*
SCHIZOPHRENIA	DDE	1	mim:181500	0.152	*APOL2*
SCHIZOPHRENIA	opDDT	1	mim:181500	0.249	*APOL2*
SCHIZOPHRENIA	DDT	1	mim:181500	0.273	*APOL2*
SEX REVERSAL	DDE	1	mim:300018	0.00628	*NR0B1*
SEX REVERSAL	opDDT	1	mim:300018	0.0109	*NR0B1*
SEX REVERSAL	DDT	1	mim:300018	0.0121	*NR0B1*
SOTOS SYNDROME	DDE	1	mim:117550	0.00628	*NSD1*
SOTOS SYNDROME	opDDT	1	mim:117550	0.0109	*NSD1*
SOTOS SYNDROME	DDT	1	mim:117550	0.0121	*NSD1*
TESTICULAR CANCER	DDT	10	mesh :d013736	0.0037668	*CYP3A4;GJA1;JUN;MT2A;ABCB1;MAPK1;MAPK3;ZFP36L1;CASP3;NR1I2*

Supplementary Material, Table 2 (continued)

DISEASE	CHEMICAL	NUMBER OF GENES	SOURCE	P-VAL	*LIST OF GENES*
TESTICULAR CANCER	DDE	1	mim:273300	0.0125	*KIT*
THYROID CANCER	DDT	45	mesh :d013964	0.00046698	*CFTR;PNRC1;DUSP10;MAPK14;CYP3A4;CYP11A1;TSC22D3;DUSP1; DUSP3;DUSP6;NR0B1;AHR;ERF;ESR1;ETV5;FOS;GJA1;HAL;HSD17B1; IL1B;IL2;AR;JUN;NFKB1;NRD1;NTRK3;PGR;ABCB1;PLA2G2A;POU4F1; POU4F2;MAPK1;MAPK3;MAPK8;MAPK9;RAGE;RXRB;TNF;TSHB;TSHR; CASP3;PLA2G10;CASP7;NR0B2;NR1I2*
THYROID CANCER	DDE	1	mim:188550	0.0432	*NCOA4*
THYROID CANCER	opDDT	1	mim:188550	0.0739	*NCOA4*
THYROID CANCER	DDT	1	mim:188550	0.0821	*NCOA4*
TYPE II DIABETES	DDT	40	mesh_d003924	1.22E-05	*CFTR;PNRC1;MAPK14;CYP3A4;CYP11A1;TSC22D3;DUSP1;AHR;ESR1; ESR2;FOS;FOSL2;GJA1;HAL;HSD17B1;HSPA5;IL1B;IL2;AR;JUN;JUNB; MT2A;NFKB1;PGR;ABCB1;PLA2G1B;POMC;MAPK1;MAPK3;MAPK8; SCN8A;NFKBIZ;ZFP36L1;TNF;TSHB;UGT2B7;CASP3;NR0B2;TNFSF11; NR1I2*
WEAVER SYNDROME	DDE	1	mim:277590	0.00628	*NSD1*
WEAVER SYNDROME	opDDT	1	mim:277590	0.0109	*NSD1*
WEAVER SYNDROME	DDT	1	mim:277590	0.0121	*NSD1*
